# Antidiabetic Micro-/Nanoaggregates from Ge-Gen-Qin-Lian-Tang Decoction Increase Absorption of Baicalin and Cellular Antioxidant Activity In Vitro

**DOI:** 10.1155/2017/9217912

**Published:** 2017-07-17

**Authors:** Dai Lin, Qian Du, Huiqin Wang, Guanzhen Gao, Jianwu Zhou, Lijing Ke, Tianbao Chen, Chris Shaw, Pingfan Rao

**Affiliations:** ^1^Food Nutrition Research Centre, School of Food Science and Biotechnology, Zhejiang Gongshang University, Hangzhou, Zhejiang 310012, China; ^2^Institute of Biotechnology, Fuzhou University, Fuzhou, Fujian 350002, China; ^3^Natural Drug Discovery Group, School of Pharmacy, Queen's University Belfast, 97 Lisburn Road, Belfast, UK

## Abstract

The antidiabetic effects of Ge-Gen-Qin-Lian-Tang decoction (GQD) have been proven clinically. In a pharmacological study conducted on STZ-induced diabetic rats, the constitutive aggregates/sediments of Ge-Gen-Qin-Lian-Tang decoction exhibited stronger hypoglycemic and antioxidant activities compared to the soluble compositions. This study aims to demonstrate the pharmacological properties of aggregates derived from GQD by measuring permeability of the active monomer phytochemicals (e.g., baicalin) in a Caco-2 cell monolayer and determine the cellular viability, intracellular redox status (MDA and SOD), and insulin secretion of pancreatic *β*-cell line, INS-1, following STZ-induced oxidative stress. The aggregates were separated into three fractions, namely, “MA (microaggregates),” “400 g supernatant,” and “MNA (micro-/nanoaggregates),” by centrifugation at 400 ×g and 15000 ×g, respectively. Aggregates in the sediment increased baicalin absorption, showed little toxicity to *β*-cells, elevated intracellular SOD levels, and significantly suppressed oxidative damage effects on cellular viability and functions. The “MA” fraction had a larger particle size and provided higher antioxidant cellular protection than “MNA” in vitro, implying that the sediments may be the active components in the herbal decoction. The actions of these micro-/nanoaggregates may provide a new perspective for understanding the antidiabetic effects of herbal decoctions and aid in interpretation of synergistic actions between the multiple components.

## 1. Introduction

Herbal decoctions from traditional Chinese medicine (TCM) have always been an option for treating oxidative stress-related chronic diseases [1–3], including diabetes. Many active components have been identified from various medicinal plants, for example, flavonoids [4], polyphenols [5], and organic acids and alkaloids, polypeptides, and polysaccharides [1, 6]. These components may work alone as a single chemical compound or, in many instances, may deliver much more potent therapeutic effects in synergy with other components from the same plant or in a combination of different herbs, as demonstrated in many herbal TCM studies [7–9]. Besides the assumption that multiple components may be able to regulate several molecular pathways simultaneously, the rich content of colloidal particles and aggregates formed by various components in a decoction has been demonstrated in TCM (Ma-Xing-Shi-Gan-Tang). In one such instance, ephedrine and pseudoephedrine were mostly found to be bound to colloidal nanoparticles which changed their bioactivities [10] and this effect may provide the supramolecular structures for these synergistic actions.

As a classic herbal TCM dating back to the East Han Dynasty, Ge-Gen-Qin-Lian-Tang decoction (GQD) is prepared from a formula composed of Radix Puerariae Lobatae, Radix Scutellariae, Rhizoma Coptidis (Chinese goldthread), Radix Glycyrrhizae (licorice, honey-processed), and* Zingiber officinale* Roscoe (ginger) and has been used for the clinical treatments of type 2 diabetes and intestinal inflammation [11, 12]. GQD significantly reduced glycated hemoglobin (HbA1c) and fasting blood glucose (FBG) in streptozotocin- (STZ-) and high-fat-diet-induced diabetic SD rats and enhanced glucose consumption in 3T3-L1 adipocytes [13]. In T2D patients, GQD significantly reduced HbA1c, while regulating the ecological structure of the gut microbiota by enriching the amounts of beneficial bacteria, indicating that the gut is among the important biological targets of this herbal tonic [12].

GQD contains several well-known active phytochemicals, that is, baicalin [14, 15], berberine [16, 17], puerarin [18], glycyrrhizic acid, and liquiritin [19], which are correlated to the antidiabetic, antioxidant, and immunoregulative effects. Apart from its antihyperglycemic and antioxidant activities, baicalin is also known as a prolyl endopeptidase inhibitor which induces apoptosis in pancreatic cancer cells [20, 21] and it also induces apoptosis of lymphoma cells by downregulation of the PI3K/Akt signaling pathway [22]. However, taking the plant-derived flavone baicalin as an example, poor solubility and bioavailability are a common problem of these phytochemicals when applied clinically, resulting in the efficacy and pharmaceutical mechanisms of TCM decoctions being often questioned.

In our previous studies, GQD was found to exert antihyperglycemic effects on streptozocin- (STZ-) induced diabetic rats, while the micro-/nanoaggregates (sediments produced after high speed centrifugation) showed stronger activities than the supernatant on lowering blood glucose levels, elevating fasting blood insulin and insulin secretion index and increasing SOD activity of liver and pancreas [23]. These data implied that the micro-/nanoaggregates of TCM decoctions may account for their therapeutic effects, being at least as effective as the soluble components. The rich content of insoluble small molecules and self-assembled colloidal particles in these decoctions may account for the formation of micro-/nanometer scale aggregates by secondary aggregation. The properties and functions of these aggregates warrant careful and systematic study.

To understand the cellular functions and antihyperglycemic mechanisms of aggregates in GQD, their particulate characteristics and effects on cell proliferation, insulin secretion, and redox status of pancreatic *β*-cells were assessed together with their impact on absorption of baicalin across Caco-2 colonic epithelial cell monolayers in vitro.

## 2. Materials and Methods

### 2.1. Materials

The herbs used in this study, for example, Radix Puerariae Lobatae, Radix Scutellariae, Rhizoma Coptidis (Chinese goldthread), Radix Glycyrrhizae (licorice, honey-processed), and sun-dried roots of* Zingiber officinale* Roscoe (ginger) were purchased from Beijing Yanjing Chinese Medicinal Herbs Co. Ltd. and authenticated by Professor Chengzi Yang from Fujian University of Traditional Chinese Medicine.

Cell culture medium (RPMI-1640, DMEM), HBSS buffer, penicillin-streptomycin solution, and NEAA were from HyClone (Xiamen, China); Fetal Bovine Serum (FBS) was from Biological Industries (BIOIND); HEHPE, 0.25% Trypsin-EDTA, L-glutathione (reduced), and MTT were from AMRESCO Co. Ltd. (USA); streptozocin (STZ), sodium bicarbonate, and sodium pyruvate were from Sigma (USA). Baicalin was purchased from the Fujian Institute for Food and Drug Quality Control. Malondialdehyde (MDA), superoxide dismutase (SOD), and bicinchoninic acid (BCA) kits were from Nanjing Jiancheng Biotechnology Institute; ELISA kits for insulin measurements were purchased from Yanyu Biotech (Shanghai) Co. Ltd.

### 2.2. Preparation of GQD, MA, and MNA

Ge-Gen-Qin-Lian-Tang decoction (GQD) was prepared by soaking Radix Puerariae Lobatae 72 g, Radix Scutellariae 27 g, Rhizoma Coptidis 27 g, Radix Glycyrrhizae 18 g, and ginger 4.5 g in 1.2 L deionized water for 30 min at 25°C and then boiled for 40 min, cooled to room temperature, and filtered through two layers of cotton gauze. The filtrate was named GQD. After being centrifuged at 400 ×g for 5 min, the sediment of GQD was collected and resuspended with cell culture medium (the same volume as GQD) and named “microaggregates (MA),” while the supernatant was collected and named “400 g supernatant.” Part of the supernatant was centrifuged at 15000 ×g for 15 min. The sediment was collected and resuspended to obtain the sample named “micro-/nanoaggregates (MNA).”

### 2.3. Determination of Particle Size

The hydrodynamic diameter of particles was determined by Dynamic Light Scattering (DLS) analysis on a Zetasizer Nano device (Malvern Instruments, Worcestershire, UK).

### 2.4. Cell Culture and MTT Assay

Human colonic epithelial cells, Caco-2 (8 × 10^4^ cells/mL, 200 *μ*L/well), and rat pancreatic *β*-cell line, INS-1 (3 × 10^5^ cells/mL, 200 *μ*L/well), were cultured at 37°C under 5% CO_2_ and 95% relative humidity for 24 h and used to evaluate the influence of GQD and aggregates on cellular viability and proliferation using the MTT assay. Samples were adjusted to the universal serial concentrations in terms of dry weight of herbs (0.98, 1.95, 3.91, 7.81, 15.63, 31.25, 62.5, and 125 mg/mL), added to the cells in 96-well plates (200 *μ*L/well), and cultured for 12 h with 5 replicates for each concentration. The test was repeated 3 times. The cell survival rate was calculated with the following equation (mean ± SD, *n* = 5):

(1) $$\begin{array}{c} \text{survival rate\%}=\frac{A_{570\;nm}\;\;sample}{A_{570\;nm}\;\;control}\times 100\%. \end{array}$$

### 2.5. Permeability Tests on Caco-2 Cell Monolayers

Baicalin concentrations were determined by an RPLC method as previously reported [24]. An RPLC column, Daisogel-C18 (5 *μ*m, 4.6 × 250 mm), was used with methanol-water-phosphoric acid (47 : 53 : 0.2) as eluting buffer, flow rate of 1.0 mL/min, monitored at 280 nm; column temperature was set to 40°C.

Permeability of GQD and its fractions was determined following a previously described protocol [25]. Briefly, the cells were seeded onto Transwell plates and allowed to form a confluent monolayer over 20 days prior to the experiment. On day 21, the test samples (0.4 mL), namely, GQD, MA, and MNA, were added to the apical side of the membrane and 0.6 mL HBSS buffer was added to the basolateral side. The transport of baicalin across the monolayer was monitored over a 3-hour time period at 37°C under 5% CO_2_. Samples (100 *μ*L) were collected from the BL side at 30, 60, 90, 120, and 180 min. HBSS buffer (100 *μ*L) was added to the BL side each time the sample was collected. MA and MNA were dispersed evenly in cell culture medium by vortexing.

The permeability coefficient (*P*_app_) and absorption rate (*A*%) were calculated from the following equations (*n* = 3):

(2) Papp=dQ/dtA×C0A%=100×QC0×V,

where the unit of *P*_app_ is cm/s, *dQ*/*dt* is the rate of permeation of the drug across the cells (*μ*g/s), *A* is the area of cell monolayer, *C*_0_ is the donor compartment concentration at time zero (*μ*g/mL), and *Q* is the total concentration of drug transfer across the cell monolayer (*μ*g). *C*_0_ is obtained from analysis of the dosing solution at the start of the experiment. *V* is the volume of donor compartment.

### 2.6. Effects of GQD, MA, and MNA on STZ-Induced Cellular Damage

INS-1 cells were seeded into 96-well plates, grown for 24 h to form a confluent monolayer, and washed with PBS. GQD, MA, and MNA (each 100 *μ*L) were added with 100 *μ*L STZ (IC_50_) and incubated for 12 h prior to MTT assay. Five duplicates were used for each sample. The test was repeated 3 times. The cell survival rate was calculated with (1) and the protection rate was calculated with the following equation (means ± SD, *n* = 5):

(3) $$\begin{array}{c} \text{protection rate\%}=\frac{\left({A_{570}}_{\text{sample}}-{A_{570}}_{\text{STZ}}\right)}{\left({A_{570}}_{\text{Normal}}-{A_{570}}_{\text{STZ}}\right)}\times 100\%. \end{array}$$

### 2.7. Effects of GQD, MA, and MNA on MDA, SOD, and Insulin Secretion

INS-1 cells (5.5 × 10^6^ cells) were seeded into 12-well plates and grown for 24 h to form a confluent monolayer. GQD, MA, and MNA (each 500 *μ*L) were added with 500 *μ*L STZ (IC_50_ = 46.4 mM) and incubated for 12 h. Cells were then washed with 1 mL KRBB prior to the addition of 3.3 mM glucose (dissolved in KRBB) and incubated for 1 h. The culture supernatants (500 *μ*L) were collected and centrifuged at 4°C and 200 ×g for 10 min and then stored at −20°C. The remaining KRBB was removed from cells before 16.7 mM glucose (dissolved in KRBB) was gently added and incubated for 1 h. The supernatant collection was then repeated as above. The cells were digested with pancreatin until 500 *μ*L of culture medium was added to stop the digestion. The cell suspension was moved to 1.5 mL tubes and centrifuged (4°C, 200 ×g, 10 min) to collect cells for MDA, SOD, insulin (INS), and protein (bicinchoninic acid) assays.

### 2.8. Statistical Analysis

The raw data were processed with EXCEL (Microsoft, Inc.) and SigmaPlot (Systat Software, Inc.), and significance levels were determined by a one-way ANOVA and indicated as *P* < 0.05 or *P* < 0.01.

## 3. Results and Discussion

### 3.1. Particle Size Distribution of GQD Aggregates

**Figure 1:**
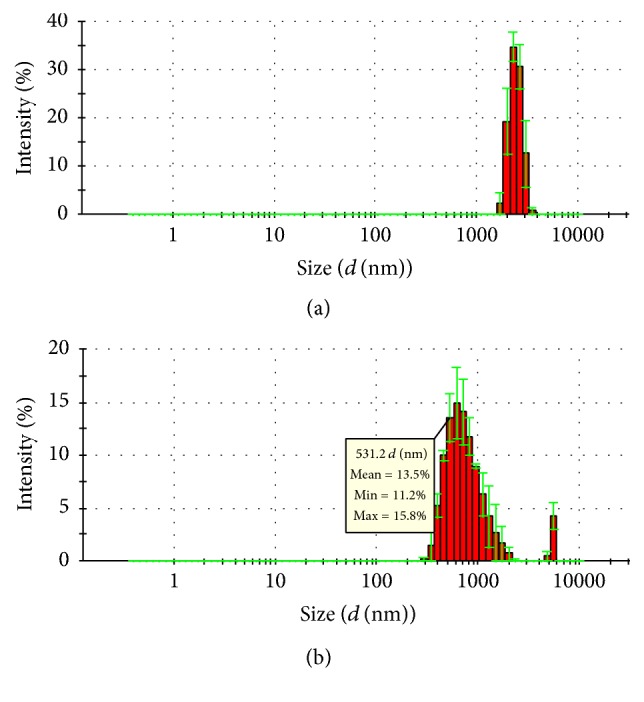
The particle size distribution of aggregates in GQD. (a) Particle size distribution of MA; (b) particle size distribution of MNA. Three duplicates were performed for each sample.

GQD was separated into three fractions by centrifugation. As shown in Figure 1, the aggregates in resuspended sediment produced after low speed centrifugation (400 ×g) had an average diameter of 2~3 *μ*m. The remaining particles in the supernatant were further separated with high speed centrifugation (15,000 ×g) and resuspended to obtain a colloidal suspension with particles having an average diameter around 530 nm (Table 1) and a major size distribution from 300 nm to 1000 nm. This centrifugation primarily separated the aggregates according to their average size and relative density, although some small colloidal particles may still remain in the supernatant of the 15,000 ×g centrifugation.

### 3.2. Increased Baicalin Absorption

As demonstrated by Lin et al. [28], hydrophobic phytochemicals, that is, baicalin, puerarin, and berberine hydrochloride, are dispersed with the assistance of components from the constituent herbs, resulting in elevated solubility. In combined use with berberine, puerarin, glycyrrhizic acid, and liquiritin, the solubility and absorption of baicalin were improved [29, 30]. As a purified component, baicalin is barely soluble in aqueous solution and has a very poor absorption rate of only 1% [26, 27].

In this study, the intestinal absorption of baicalin from GQD was assessed in a Caco-2 cell monolayer model for evaluating whether formation of aggregates altered the bioavailability of Ge-Gen-Qin-Lian-Tang decoction (GQD). The baicalin-containing GQD showed 5-fold higher *P*_app_ than baicalin alone (Table 2). MA contained 48% of decocting baicalin, exhibiting nearly twice *P*_app_ and absorption rate in comparison to GQD. Meanwhile, MNA representing approx. 46% of total baicalin in the decoction exhibited highest *P*_app_ and absorption rate (Table 2). It is quite clear that the inclusion of baicalin in the sediments, even in terms of micrometer-scaled aggregates, strongly assisted transportation of Ge-Gen-Qin-Lian-Tang decoction (GQD) across the Caco-2 cell monolayers, significantly increasing its absorption.

**Figure 2:**
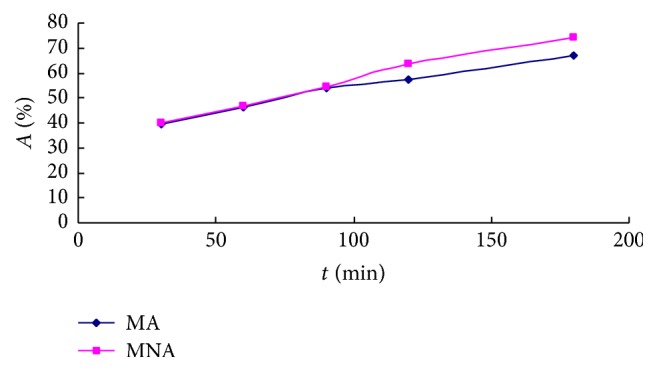
Absorption rate (*A*%) of baicalin in GQD aggregates on monolayers of Caco-2 cells. Baicalin concentrations in the basolateral side solutions were determined by HPLC at different time points (*n* = 4).

The absorption rate (*A*%) of baicalin in GQD aggregates across the Caco-2 cells monolayer (from apical side to basolateral side) was determined at 30, 60, 90, 120, and 180 min of incubation, as shown in Figure 2. Within the first 90 min, the baicalin absorption rates of MA and MNA were the same. After incubation for a longer time (2 h and 3 h), the MNA exhibited an 8% higher absorption than the MA, indicating that smaller particles may act as the more efficient vehicle for baicalin. Meanwhile, *A*% of baicalin in GQD were 19% at 30 min and 35% at 3 h, which were lower than those of aggregates but higher than those of baicalin alone, implying a significantly improved absorption in the herbal suspension compared to the pure baicalin solution.

It is well known that the glycyrrhizic acid (a licorice-derived glycoside) is capable of forming intermolecular complexes to increase the solubility of poorly soluble drugs [31]. Our earlier work has also shown that even aqueous soluble plant-derived alkaloids (ephedrine) were mainly carried by colloidal nanoparticles self-assembled in another TCM herbal decoction and therefore exhibited different pharmacological characteristics from own monomer of Ge-Gen-Qin-Lian-Tang decoction (GQD) [10]. As demonstrated above, higher *P*_app_ and absorption rates of GQD aggregates indicate that the inclusion of baicalin in the higher order structures (i.e., supermolecular complexes and aggregates) changes pharmacokinetics of Ge-Gen-Qin-Lian-Tang decoction (GQD) and may be essential for its synergistic actions in the herbal decoction. Such complexes could be formed with flavonoids (such as puerarin and liquiritin), alkaloids (such as berberine), glycosides (such as baicalin and glycyrrhizic acid), polysaccharides, and glycated proteins.

### 3.3. Influence of GQD and Its Aggregates on INS-1 Pancreatic *β*-Cell Proliferation

**Figure 3:**
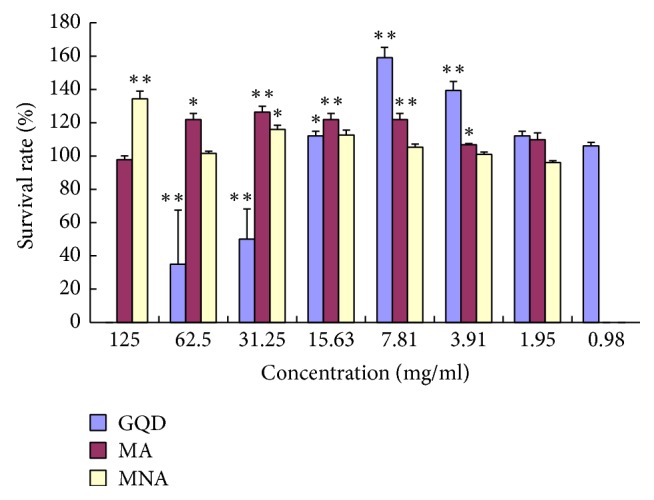
Effects of GQD and its aggregates on proliferation of INS-1 pancreatic *β*-cells. *n* = 5. GQD (in blue): compared with normal controls, 0.98~1.95 mg/mL (*P* > 0.05), 15.63 mg/mL (0.01 < *P* < 0.05, “*∗*”), and others (*P* < 0.01, “*∗∗*”); MA (in orange): 400 g sediment, compared with normal controls, 125.0 mg/mL and 1.95 mg/mL (*P* > 0.05), 62.50 and 3.91 mg/mL (0.01 < *P* < 0.05, “*∗*”), and others (*P* < 0.01, “*∗∗*”); MNA (in grey): 15000 g sediment, compared with normal controls, 125.0 mg/mL (*P* < 0.01, “*∗∗*”), 31.25 mg/mL (0.01 < *P* < 0.05, “*∗*”), and others (*P* > 0.05). Error bars + SEM. Differences are significant according to a one-way ANOVA indicated with an asterisk (*P* < 0.05; *n* = 4) or double asterisks (*P* < 0.01; *n* = 4).

As shown in Figure 3, GQD suppressed the growth of INS-1 pancreatic *β*-cells at 31.25~62.50 mg/mL, implying a significant cytotoxicity (*P* < 0.01). However, at lower concentrations (15.63 mg/mL and lower), GQD showed no inhibition on the cell proliferation but rather promotion of such (max. 60% at 7.81 mg/mL). In contrast, the aggregates, both of MA and MNA, showed no cytotoxicity on INS-1 cells at concentrations as high as 125 mg/mL. This indicates that most of the cytotoxic compositions of GQD are in the supernatant after high speed centrifugation, which contains the majority of aqueous solutes. At medium concentrations (7.81~62.5 mg/mL), both MA and MNA mildly promoted cell proliferation, while the larger sized aggregates (MA) exhibited slightly higher proliferation rates, that is, 28% at 31.25 mg/mL. The proliferation promoting activities of GQD and its aggregates may be attributed to their intracellular antioxidant capacities, since the pancreatic *β*-cells are sensitive to oxidative stress.

The effective concentration of GQD and its fractions appeared to be very high (in milligrams). It is because the concentration was presented in terms of the total dry weights of herbal materials used in preparing GQD. Given that the decocting only extracts a small portion of herbal materials, the dry weight of actual GQD dispersion and its aggregate fractions would be many times lower.

### 3.4. Inhibition of STZ-Induced Cellular Oxidation

**Figure 4:**
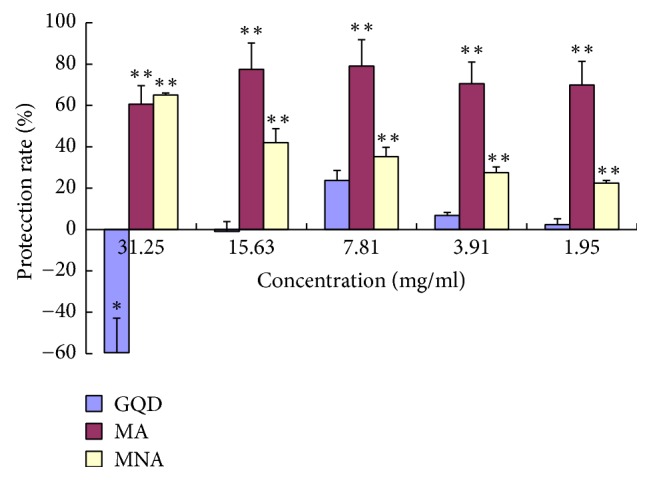
Protection of GQD and its aggregates against STZ-induced oxidative suppression of the growth of INS-1 *β*-cells. *n* = 5. Oxidative damage was induced with STZ at its IC_50_ (46.4 mM). GQD: compared with STZ controls, 31.25 mg/mL (0.01 < *P* < 0.05, labelled “*∗*”) and others (*P* > 0.05); MA: 400 g sediment, compared with STZ controls, at all concentrations, *P* < 0.01 (“*∗∗*”); MNA: 15000 g sediment, compared with STZ controls, at all concentrations, *P* < 0.01 (“*∗∗*”).

As shown in Figure 4, GQD protected INS-1 cells from STZ-induced oxidative damage by 23% at 7.81 mg/mL but showed no protection at higher or lower concentrations. In comparison, both MA and MNA significantly protected the cells at a much wider range of concentrations (1.95 to 31.25 mg/mL) and achieved much stronger protection (MA, 78% at 7.81 mg/mL). It indicates that antioxidants or components capable of elevating the cellular antioxidant capacity are embedded in the aggregates but not in the soluble fraction. Given that neither MA nor MNA exhibited cytotoxicity (Figure 3), the cytotoxic components of GQD are most likely to be in the supernatant. Meanwhile, the larger sized aggregates generally showed significantly higher protection rates (*P* < 0.01) than the smaller sized aggregates. Notably, the protection rates of MA were irrelevant to dosage, while those of MNA were dose-dependent, indicating that these two groups of aggregates may work via different mechanisms to inhibit cellular oxidative damage.

The significantly elevated MDA level (Table 3) and reduced SOD level (Table 4) indicated that INS-1 cells had been damaged by STZ-induced oxidation. The aggregates (both MA and MNA) significantly restored the cellular SOD activity and reduced the MDA level, whereas GQD only exhibited significant antioxidant effects at 7.81 mg/mL. This is consistent with the different performance of the aggregates and GQD on regulating cellular viability (Figure 4). By comparing the MAD and SOD levels of GQD and its fractions, their antioxidant activities were ranked in sequence: MA > 400 g supernatant > MNA > GQD.

The supernatant is rather high in antioxidant activity but is toxic to the cells, implying that, at high GQD concentrations, the cytotoxic components overrule the antioxidant (cytoprotective) components and therefore kill the cells.

### 3.5. Restoration of Insulin Secretion

**Figure 5:**
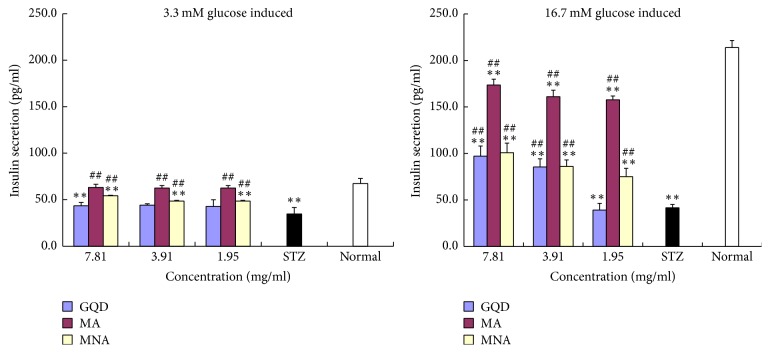
GQD and its aggregates restoration of insulin secretion in STZ-damaged INS-1 cells. “*∗∗*”: compared with normal cells, *P* < 0.01, *n* = 3; “##”: compared with STZ controls, *P* < 0.01, *n* = 3.

**Figure 6:**
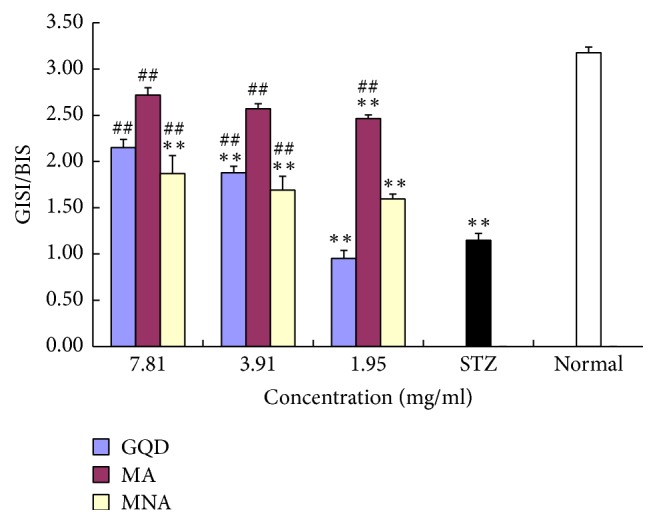
GQD and its constitutive aggregates elevation of insulin secretion index (ISI) of STZ-damaged INS-1 *β*-cells. “*∗∗*”: compared with normal cells, *P* < 0.01, *n* = 3; “##”: compared with STZ controls, *P* < 0.01, *n* = 3; ISI: GSIS/BIS.

The impacts of STZ-induced oxidation and GQD samples on insulin secretion from pancreatic *β*-cells were evaluated at either baseline levels (3.3 mM) or stimulated levels (16.7 mM) of glucose, as shown in Figure 5. The insulin secretion index (ISI) was calculated as a ratio of glucose-stimulated insulin secretion (GSIS)/basal insulin secretion (BIS) and data are shown in Figure 6.

STZ-induced oxidation reduced the expression and secretion of insulin, causing cells to be irresponsive to the glucose stimulus. The baseline insulin secretion of normal INS-1 *β*-cells was 67 pg/mL, which was dramatically increased threefold to the stimulated level of 214 pg/mL (ISI = 3.23). In contrast, STZ-damaged cells did not respond to such a stimulus. The presence of GQD did not improve the baseline insulin secretion of STZ-damaged cells but doubled the insulin secretion (max. 97 pg/mL, *P* < 0.01) at corresponding elevated glucose levels.

Aggregates from GQD, namely MA and MNA, significantly improved both the baseline and stimulated insulin secretions (*P* < 0.01, Figure 5). Notably, the larger size aggregates (MA) showed much stronger restorative power than the MNA, wherein the BIS was almost fully restored and the GSIS (max. 174 pg/mL, ISI = 2.7) was about twofold higher than that of MNA. The MNA significantly improved the GSIS (max. ISI = 1.9) in a dose-dependent manner. Its overall effects were rather like GQD, except that the effective concentration of MNA was lower than that of GQD.

All the insulin secretion results were consistent with the cellular protection and antioxidant effects of GQD and its constitutive aggregates. The significant higher antioxidant activity of aggregates on cells was in good agreement with their antioxidant effects in vivo [23], wherein the aggregates elevated SOD levels in pancreas, kidney, and liver of STZ-induced diabetic rats. Despite the higher bioavailability of baicalin in MNA demonstrated earlier in this study, the aggregates in MA showed more potent protective effects against STZ-induced oxidative stress upon cells. Although it remains unclear why larger size aggregation particles exhibited stronger antioxidant activity, one can anticipate that such aggregates may have a higher content of free radical scavenging compounds, such as berberine, puerarin, liquiritin, and glycyrrhizin acid [15–19, 32]. Besides, the MA is still a high absorption drug, whose absorption rate was only 7% lower than that of MNA. The slightly lower absorption rate could be compensated by the richer contents of particles in MA indicated by their higher scattering light intensity (kcps).

Although the higher absorption rate and antioxidant and cellular protective activities in vitro do not necessarily mean better therapeutic effectiveness in vivo, it is reasonable to anticipate that the micro-/nanoscale aggregates may have a vital contribution to the overall antidiabetic effects of the herbal decoction (GQD), noting that the herbal components would eventually interact with the mammalian digestive tract in the form of multiple-order aggregates, such as chyle.

## 4. Conclusions

The antidiabetic herbal tonic, GQD, contains micro- and nanoscale aggregates which improve the bioavailability of insoluble phytochemicals, that is, baicalin, and possess little cytotoxicity on colonic epithelial cells and pancreatic *β*-cells (INS-1) in vitro. It also elevates cellular antioxidant enzymes and protects *β*-cells from STZ-induced oxidation and restores their insulin secretion capability. The centrifugal separation results in two different size distribution fractions of aggregates (centrifuge sediments), and the larger size aggregates (MA) possessed stronger protection on cellular viability and function of *β*-cells in vitro. These data are consistent with an earlier antihyperglycemic study of GQD aggregates on STZ-induced diabetic rats. The aggregates from the TCM decoction, for the first time, have been found to contain active components that contribute to the antidiabetic activity of the herbal tonic by exhibiting antioxidant effects on the endocrine cells and the carrying of insoluble compounds across the intestinal mucosal barrier. These data also imply that the aggregates and sediments in the herbal decoction should be handled with greater care for both TCM herbal medicine production and pharmacological studies.

In comparison with monomer compound studies, it would require different approaches to elucidate the pharmacological mechanisms underpinning the therapeutic actions of TCM aggregates and identify the constituent chemicals of aggregates in different size. The particulates can be further separated by ultrafiltration or size-exclusion chromatography or ion-exchange chromatography according to their physical size, for example, diameter, or surface charge. The phase extraction and/or enzymatic hydrolysis can then be applied to deconstruct these separated fractions of aggregates, whose chemical compositions will be resolved with chromatographic approach coupled with mass spectra. Thus, more comprehensive studies on these colloidal micro-/nanoparticles and their constituent compounds are warranted to fully understand their pharmacological characteristics and chemical natures, which may inspire and lead to the development of active supramolecular complexes for the treatment of oxidative diseases.

## Figures and Tables

**Table 1:** Average diameter of aggregates in centrifuged sediments of GQD.

Sample	*Z*-Average *d *(nm)	Derived count rate (kcps)
Microaggregates (MA)	2775 ± 712	331 ± 17
Micro-/nanoaggregates (MNA)	531 ± 23	241 ± 2

*n* = 3; *Z*-Average *d* (nm) is the average diameter of particles. Derived count rate (kcps) partially indicates the particle concentration. MA: the resuspended sediment of GQD after low speed centrifugation (400 ×g); MNA: the resuspended sediment of GQD obtained by high speed centrifugation (15,000 ×g). Derived count rate: the intensity of light scattered by particles, presented as “thousand counts per second (kcps).”

**Table 2:** The apparent permeability (*P*_app_) and absorption rate of baicalin across Caco-2 cell monolayers.

Sample	Apical side baicalin concentration (*μ*g/mL)	*P*_app_ (×10^−6^ cm/s)	Absorption rate in 3 h	Absorption level
Pure baicalin^*∗*^	—	0.66 ± 0.10	~1%	Low
GQD	27.6	3.40 ± 0.21	35%	Medium
MA	12.9	6.60 ± 0.18	67%	High
400 g supernatant	14.2	6.59 ± 0.29	66%	High
MNA	12.8	7.30 ± 0.17	74%	High

^*∗*^Data is cited from [26, 27]. *n* = 4. *P*_app_ > 5 × 10^−6^ cm/s: high absorption; *P*_app_  =  1~5  ×  10^−6^ cm/s: medium absorption; *P*_app_ < 1 × 10^−6^ nm/s: low absorption.

**Table 3:** GQD and components reduced STZ-induced MDA in pancreatic *β*-cell line, INS-1.

Group	MDA (nmol/mL)			
	GQD	MA	400 g supernatant	MNA
7.81 mg/mL herbs	9.40 ± 0.47^ab^	7.13 ± 1.71^ab^	8.29 ± 1.07^ab^	8.85 ± 0.77^ab^
3.91 mg/mL herbs	9.81 ± 0.24^ab^	7.41 ± 1.55^ab^	8.78 ± 0.81^ab^	9.17 ± 0.60^ab^
1.95 mg/mL herbs	10.06 ± 0.12^a^	7.49 ± 1.51^ab^	9.04 ± 0.67^ab^	9.37 ± 0.50^ab^
STZ control	10.25 ± 0.24^a^			
Normal	5.89 ± 0.17^b^			

*n* = 5; ^a^compared with normal cells (*P* < 0.05); ^b^compared with STZ controls (*P* < 0.05).

**Table 4:** GQD and components restored SOD activity in STZ-treated pancreatic *β*-cell line, INS-1.

Group	SOD (U/mg·protein)			
	GQD	MA	400 g supernatant	MNA
7.81 mg/mL herbs	83.95 ± 5.32^ab^	133.33 ± 8.02^b^	106.44 ± 4.36^ab^	93.77 ± 8.87^ab^
3.91 mg/mL herbs	66.22 ± 7.60^a^	127.40 ± 8.40^ab^	94.97 ± 5.35^ab^	85.63 ± 3.69^ab^
1.95 mg/mL herbs	62.00 ± 6.56^a^	125.33 ± 5.51^ab^	87.93 ± 6.76^ab^	80.60 ± 4.23^ab^
STZ Control	60.75 ± 4.07^a^			
Normal	155.88 ± 6.80^b^			

*n* = 5; ^a^compared with normal cells (*P* < 0.05); ^b^compared with STZ controls (*P* < 0.05).
